# Mechanism- and Physiology-Guided Drug-Coated Balloon-Only Percutaneous Coronary Intervention in Complex Multivessel Coronary Artery Disease: Insights From IVUS and vFFR

**DOI:** 10.1016/j.jscai.2026.104391

**Published:** 2026-02-19

**Authors:** Giovanni Martino, Francesco Fabio Greco, Bernardo Cortese, Giovanni Coscarelli, Ciro Indolfi, Antonio Curcio, Alberto Polimeni

**Affiliations:** a Department of Pharmacy, Health Sciences and Nutrition, University of Calabria, Arcavacata di Rende, Italy; b Division of Interventional Cardiology, "S.S. Annunziata" Hospital, Cosenza, Italy; c University Hospitals Harrington Heart & Vascular Institute, Cleveland, Ohio

**Keywords:** drug-coated balloon, intravascular ultrasound, vessel fractional flow reserve

## Case presentation

**Figure 1:**
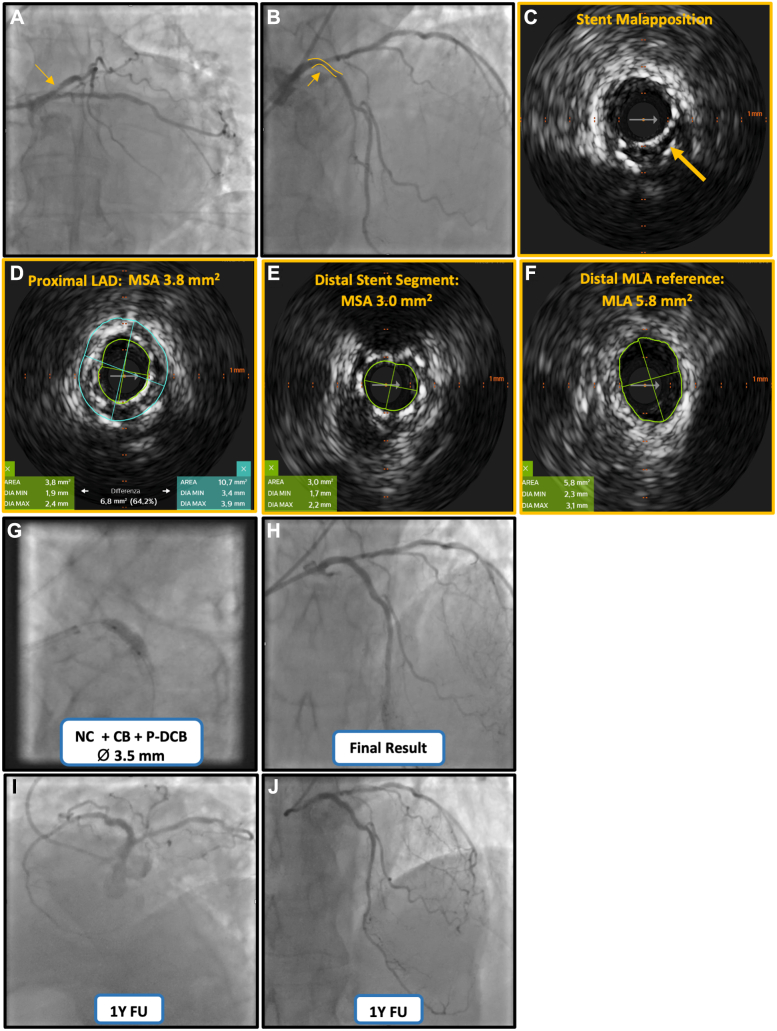
**Procedural steps of intravascular ultrasound (IVUS)-guided percutaneous coronary intervention of proximal left anterior descending artery (LAD) in-stent restenosis and 1-year angiographic Follow-up.** (**A**, **B**) Baseline LAD angiography in caudal and cranial projections, respectively. (**C**) IVUS cross-sectional frame acquired with the Eagle Eye Platinum catheter (Philips), demonstrating focal stent malapposition (yellow arrow). (**D**, **E**) IVUS frames showing minimum stent area (MSA) of 3.1 mm^2^ in the distal stent segment (**D**) and 3.8 mm^2^ in the proximal stent segment (**E**). (**F**) IVUS frame of the distal reference vessel, showing a reference minimum lumen area (MLA) of 5.8 mm^2^ with diameters D_max_ 3.1 mm and D_min_ 2.3 mm. (**G**) Mechanism-based treatment with high-pressure noncompliant (NC) balloon dilatation followed by cutting balloon (CB) angioplasty and paclitaxel drug-coated balloon (P-DCB) inflation. (**H**) Final angiographic result. (**I**, **J**) One-year angiographic follow-up (FU) demonstrating sustained patency and a favorable long-term outcome of DCB angioplasty.

**Figure 2:**
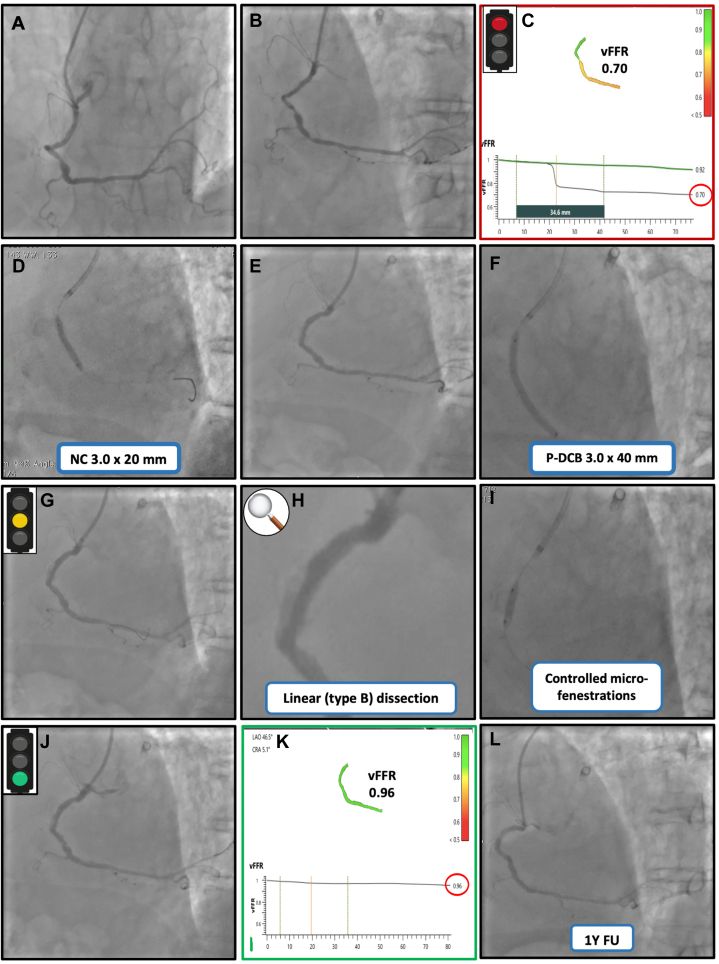
**Procedural steps of functional coronary angiography (FCA) guided percutaneous coronary intervention of the right coronary artery (RCA).** (**A**, **B**) Baseline RCA angiography in left anterior oblique and cranial projections, respectively. (**C**) FCA computed with cardiovascular angiographic analysis systems for vessel fractional flow reserve (vFFR) (Pie Medical Imaging BV) showing a functionally significant mid-RCA stenosis (vFFR 0.70). (**D**) Lesion preparation with semicompliant and noncompliant (NC) balloon dilatation. (**E**) Angiographic result after lesion preparation. (**F**) Paclitaxel drug-coated balloon (P-DCB) inflation. (**G**, **H**) Linear National Heart, Lung, and Blood Institute type B coronary dissection after DCB inflation (highlighted in light yellow). (**I**) Cutting-balloon inflation to create controlled intimal microfenestrations. (**J**) Final angiographic result. (**K**) Final vFFR assessment confirming an optimal functional result (0.96). (**L**) One-year angiographic follow-up (FU) demonstrating sustained patency and favorable long-term outcome.

We report the case of a 77-year-old man admitted with unstable angina. His medical history was notable for prior percutaneous coronary intervention (PCI) to the left anterior descending artery (LAD) in 2017, with implantation of 2 overlapping stents (2.5 × 15 mm and 2.75 × 18 mm), and diffuse large B-cell non-Hodgkin lymphoma treated within the previous 12 months. Coronary angiography revealed a significant in-stent restenosis (ISR) of the proximal LAD (Figure 1A, B), 40% stenosis of the left circumflex artery, and a long stenosis of the middle right coronary artery (RCA), visually estimated at 50% to 70% narrowed (Figure 2A, B). Given the complexity of the LAD ISR, we elected to initially address this lesion using an intravascular ultrasound (IVUS)-guided approach, with the aim of identifying the dominant restenosis mechanism and tailoring the interventional strategy accordingly.

Intravascular imaging of the proximal LAD ISR demonstrated stent underexpansion with a minimum stent area (MSA) of 3.8 mm^2^ proximally and 3.1 mm^2^ distally (Figure 1D, E), with only focal malapposition in selected frames (Figure 1C). Because the stent covered the LAD ostium, a proximal nonstented reference segment was not available; therefore, vessel sizing was derived from the first nonstented distal reference segment immediately distal to the stent (beyond the first septal branch), which showed a reference lumen area of 5.8 mm^2^ (Dmax 3.1 mm; Dmin 2.3 mm) (Figure 1F). Relative stent expansion, defined as MSA divided by the distal reference lumen area, was 65.5% (proximal MSA) and 53.4% (distal MSA), consistent with marked underexpansion and a clear mismatch between the previously implanted stent dimensions and the distal reference vessel size. The entire IVUS pullback run is provided as a Supplemental Video.

The LAD ISR was treated with high-pressure dilatation using a 3.5-mm noncompliant balloon, followed by a 3.5-mm cutting balloon and subsequent inflation of a 3.5-mm paclitaxel-coated drug-coated balloon (DCB), achieving an angiographically satisfactory result (Figure 1E, F).

To assess the hemodynamic significance of the RCA lesion, functional coronary angiography (FCA) using cardiovascular angiographic analysis systems for vessel fractional flow reserve (vFFR) (Pie Medical Imaging BV) was chosen as a rapid, wire-free physiological assessment during the index procedure. Three-dimensional (3D) quantitative coronary angiography demonstrated a lesion length of 34.5 mm, with a diameter stenosis of 49% and an area stenosis of 74%. The resulting vFFR was 0.70, well below the 0.80 cutoff and outside the gray zone, with 3D analysis showing a predominantly focal pressure-drop pattern (Figure 2C). Lesion preparation consisted of predilatation with a 3.0 × 20-mm semicompliant balloon followed by a 3.0 × 20-mm noncompliant balloon (Figure 2D), resulting in an adequate angiographic outcome (Figure 2E).

Given the patient’s high bleeding risk, a stent-free strategy was selected, consisting of prolonged inflation of a paclitaxel-coated DCB sized 1:1 to the reference vessel diameter (Figure 2F). DCB inflation resulted in a 10- to 15-mm linear National Heart, Lung, and Blood Institute (NHLBI) type B dissection, without flow limitation (Figure 2G, H).

Therefore, a 3.0 × 15-mm cutting balloon was subsequently inflated to create controlled intimal microfenestrations (Figure 2I), with the aim of decompressing the dissection plane and reducing the risk of dissection-related intramural compression and subsequent true-lumen compromise. The final angiographic result was optimal (Figure 2J) and was corroborated by an excellent functional outcome (vFFR 0.96; Figure 2K). The patient was discharged the next day in good clinical condition. Twelve-month angiographic follow-up confirmed sustained vessel patency and preservation of the procedural result (Figure 1G, H and Figure 2L).

## Discussion

This case illustrates an integrated imaging- and physiology-guided approach to PCI in a complex patient. Intravascular imaging was used to characterize the underlying mechanism of ISR in the LAD, while FCA—a wire-free, angiography-derived physiological assessment computed from routine coronary angiographic images to estimate the hemodynamic significance of a coronary stenosis—guided both the indication to treat and procedural optimization of an angiographically intermediate de novo lesion of the RCA.

ISR represents a heterogeneous pathological process, and procedural success depends on identifying the dominant underlying mechanism rather than applying a uniform treatment strategy.^1^ In the present case, IVUS provided pivotal information by demonstrating a markedly reduced MSA and significant stent underexpansion, with a mismatch between the originally implanted stent size and the reference vessel dimensions. These findings carry important procedural implications. Stent optimization in this setting typically relies on high-pressure noncompliant balloon dilatation to improve stent apposition and achieve adequate luminal expansion. Adjunctive use of a cutting balloon may facilitate controlled plaque modification and neointimal disruption, potentially enhancing luminal gain and the effectiveness of subsequent drug delivery. DCB therapy enabled local administration of an antiproliferative agent without the implantation of an additional metallic layer, an approach that is particularly appealing when further stent placement is undesirable.

The most instructive aspect of this case, however, lies in the physiology-guided management of the RCA lesion. Intermediate angiographic stenoses are well known to correlate imperfectly with myocardial ischemia, particularly in the presence of diffuse atherosclerosis or complex lesion morphology. FCA addressed this limitation by providing a lesion-specific physiological assessment without the need for pressure-wire instrumentation, identifying the RCA stenosis as functionally significant and thereby supporting revascularization. Beyond binary decision making, vFFR analysis can offer insights into the spatial distribution of pressure loss along the vessel, helping the operator distinguish predominantly focal disease from diffuse functional impairment.^2^ Importantly, postprocedural vFFR assessment provided objective confirmation of postprocedural physiological normalization, reducing residual uncertainty regarding hemodynamic significance after intervention.

Given the patient’s high bleeding risk, a stent-free strategy was deliberately favored to potentially minimize the duration and intensity of dual antiplatelet therapy. DCB-only PCI in de novo coronary lesions is increasingly considered in carefully selected scenarios; however, its success critically depends on meticulous lesion preparation, appropriate balloon sizing (1:1 to the reference vessel diameter), and adequate inflation time.^3^ A recognized procedural trade-off of balloon-based strategies is the occurrence of non–flow-limiting dissections after angioplasty and DCB deployment. In this case, a linear NHLBI type B dissection developed without angiographic evidence of flow impairment. Although many type B dissections can be safely managed conservatively when coronary flow is preserved,^4^ we elected to perform cutting-balloon inflation to create controlled intimal microfenestrations. The procedural intent was to decompress the dissection plane and reduce the risk of dissection progression and subsequent true-lumen compromise; enhanced drug transfer into the vessel wall may represent an additional potential benefit. Similar “fenestration” concepts have been described in related interventional settings,^5^ although evidence specifically addressing their role in post-DCB dissections remains limited, and such an approach should therefore be individualized. Importantly, this maneuver is not guideline-driven and should be regarded as case-specific and hypothesis-generating. Additionally, we acknowledge that, without intravascular imaging, the severity and extent of the dissection and any possible intramural involvement cannot be definitively confirmed, which should be considered a limitation of the present case.

Overall, this case underscores the complementary roles of intravascular imaging and coronary physiology in contemporary PCI. IVUS enables mechanistic understanding and guides lesion-specific mechanical optimization, whereas FCA supports target-lesion selection beyond angiography and provides objective verification of procedural success.

In the present case, the postprocedural improvement in vFFR served to document physiological normalization after intervention and reduce residual hemodynamic uncertainty. Although a favorable midterm angiographic result was also observed, the post-PCI vFFR finding should be interpreted in this single-case context primarily as procedural confirmation rather than as definitive prognostic inference.

**Focus Points**

• Intravascular imaging defines the mechanism and severity of ISR and guides the optimal treatment strategy.
• Drug-coated balloons enable stent-sparing revascularization, a viable option when a shorter and less intensive regimen of dual antiplatelet therapy is desired.
• FCA offers wire-free physiological assessment to guide revascularization decisions and to objectively confirm procedural success.

## Declaration of competing interest

The authors declared no potential conflicts of interest with respect to the research, authorship, and/or publication of this article.

## Footnotes

To access the supplementary material accompanying this article, visit the online version of the *Journal of the Society for Cardiovascular Angiography & Interventions* at 10.1016/j.jscai.2026.104391.
